# A Rare Case of Hereditary Hemorrhagic Telangiectasia: A Case Report

**DOI:** 10.7759/cureus.24517

**Published:** 2022-04-27

**Authors:** Ahmad R. Khan, Salma Waqar, Muhammad Hayyan Wazir, Amina Arif

**Affiliations:** 1 Internal Medicine, Hayatabad Medical Complex Peshawar, Peshawar, PAK

**Keywords:** melena, recurrent gi bleeding, pulmonary avm, hereditary hemorrhagic telangiectasis, osler-weber-rendu syndrome

## Abstract

Hereditary hemorrhagic telangiectasia (HHT), also known as Osler-Weber-Rendu syndrome, is a very rare autosomal dominant genetic disorder that leads to abnormal blood vessel formation in the skin, mucus membranes (called telangiectasia), and organs such as the lung, liver, and brain. It occurs due to a mutation in one of the *ACVRL1*, *ENG*, and *SMAD4* genes, which code for the formation of blood vessels. The most common symptom is recurring nosebleed (epistaxis; due to rupture of nasal mucosal telangiectasia), which begins in childhood and affects about 90-95% of people with HHT. Other common signs and symptoms include punctate, linear, or splinter-like telangiectasias on the upper body, oral mucosa, or nail beds, gastrointestinal bleeding, and iron deficiency anemia. The diagnostic criteria currently in use are the Curaçao criteria. The diagnosis is made by clinical screening (e.g., history and physical exam), baseline investigations (complete blood count, hemoglobin, hematocrit, and ferritin level), genetic testing, and detailed medical imaging to detect visceral arteriovenous malformations (AVMs) such as esophagogastroduodenoscopy, colonoscopy, multiphase contrast CT, computed tomography angiography (CTA or CT Angio), magnetic resonance angiography (MRA), chest X-ray, Doppler ultrasonography, liver biopsy, and cerebral angiography. Management includes intravenous iron therapy or blood transfusion, antifibrinolytics (e.g tranexamic acid), ablation therapies (e.g. laser treatment, radiofrequency ablation, electrosurgery, sclerotherapy, and argon plasma coagulation), and systemic anti-angiogenic agents (e.g. thalidomide, bevacizumab). In this report, we present the case of a 22-year-old man from Swabi, Pakistan, with a history of recurrent epistaxis (nosebleed) from childhood, who presented with multiple episodes of melena (blood in stool), fatigue, palpitation, and iron deficiency anemia for five years. Multiple esophagogastroduodenoscopies (OGDs) and colonoscopies were done over the years, which showed AVM in the antrum and fundus of the stomach, duodenum, and colon, and a diagnosis of HHT was made. CTA and exploratory laparotomy showed ileal loop hemangiomas. He was managed with multiple blood transfusions, argon plasma coagulation (APC) for the AVMs, oral thalidomide, and steroids. Despite therapy, the patient had intermittent episodes of blood in stool and low blood counts. During his stay in Hayatabad Medical Complex (HMC), the patient was managed with high-frequency blood transfusion and bevacizumab (systemic anti-angiogenic agent). A dramatic reduction in the number of required transfusions and improvement in the patient's bloodlines and symptoms was noted. This case highlights the importance of endoscopic methods for the timely diagnosis of HHT and its management with intravenous bevacizumab.

## Introduction

The Osler-Weber-Rendu syndrome, also known as hereditary hemorrhagic telangiectasia (HHT), is a rare autosomal dominant genetic illness that causes aberrant vasculogenesis in the skin, mucus membranes, and organs such as the liver, lungs, and brain [1-2].

Epistaxis, acute and chronic gastrointestinal (GI) bleeding, and a variety of other issues are common side effects related to the involvement of other organs. Treatment focuses on minimizing bleeding from blood vessel abnormalities and, in certain cases, removing arteriovenous malformations in organs through surgery or other targeted procedures. Chronic bleeding needs iron supplements and, on rare occasions, blood transfusions. HHT is an autosomal dominant disorder that affects one out of every 5,000-8,000 people in North America [2].

The presence of at least three of the four primary clinical symptoms of HHT is required for a clinical diagnosis: epistaxis, cutaneous or mucosal telangiectases, visceral involvement, and familial history of the disease [3]. Internal organs, particularly the lungs, gastrointestinal tract, and liver, are also affected by AVMs. Cerebral involvement is found in up to 15% of cases [4].

ENG (endoglin), ACVRL1 (activin receptor-like kinase 1), SMAD4 (mothers against decapentaplegic homolog 4), and GDF2 are four main genes recently identified in the underlying mechanism of HHT (growth differentiation factor 2) [5]. Transforming growth factor (TGF)-beta signaling pathways in vascular endothelial cells are disrupted by mutations in these genes, resulting in AVM development [6].

Patients with HHT should be referred to a multidisciplinary team that includes an otolaryngologist, pulmonologist, interventional radiologist, neurologist, geneticist, cardiologist, gastroenterologist, dermatology, hepatologist, and hematologist due to the systemic nature of the condition. Humidification, nasal lubricants, hemostatic products, laser ablation, sclerotherapy, nasal closure, and oral or topical medications are used to treat nosebleeds while GI bleeding is treated with iron replacement therapy, surgical resection of bleeding sites, and medical therapy. Patients with symptomatic hepatic AVMs who cannot be controlled medically should consider liver transplantation.

## Case presentation

A 26-year-old male, normoglycemic, normotensive patient with no previous co-morbidities and a past medical history of epistaxis, fatigue, and dizziness presented to the medicine department of Hayatatabad Medical Complex (HMC) Peshawar on February 28, 2022, with dizziness, fatigue, and recurrent episodes of per-rectal bleed.

He first presented to a local facility in February 2016 for an episode of dizziness, palpitations, fatigue, and black, tarry stools for five days with a Hb level of 4, requiring multiple blood transfusions. After a period of clinical stability of three to four days, he went to Dubai. There, he was admitted four times and evaluated for pancytopenia and melena. He presented to Rashid Hospital Dubai with dizziness and black stools (two episodes/day), followed by decreased appetite.

On examination, he was vitally stable and his abdomen was soft, non-distended, and non-tender. On per-rectal examination, black stools were noted. His Hb was 6.5 g/dl with a reticulocyte count of 3.8% and a platelet count of 52,000. The patient underwent an ultrasound of the abdomen/pelvis, which was found to be normal and a presumptive diagnosis of hemorrhagic gastritis was made based on his esophagogastroduodenoscopy (EGD).

After returning to Pakistan in 2017, the patient was admitted to the department of medicine of HMC and was treated symptomatically, but continued to have episodes of melena, palpitations, and fatigue. Multiple bone marrow biopsies were done for pancytopenia, which showed peripheral destruction of platelets and megakaryocytes. Vitamin B12 and folic acid levels were reported to be normal.

In 2018, he was admitted for sudden-onset bleeding and underwent exploratory laparotomy for ileal resection and primary anastomosis for ileal loop hemangioma on a CT angiogram. The specimen was sent for histopathology, and the report showed features of cavernous hemangiomas, mild to moderate nonspecific inflammation, and moderate serositis with no evidence of granuloma or malignancy. He was scheduled for a bone scan, which was done in IRNUM Peshawar and was found to be normal. The patient underwent multiple colonoscopies during this period, which were normal and no abnormality was reported. His cytogenic study in 2020 was normal. Bone marrow biopsies, carried out multiple times for pancytopenia in 2016, 2018, and 2021, showed depleted iron stores, megaloblasts, and peripheral destruction of platelets, and thus were labeled "cytopenia of undetermined significance" by the hematologist.

**Figure 1:**
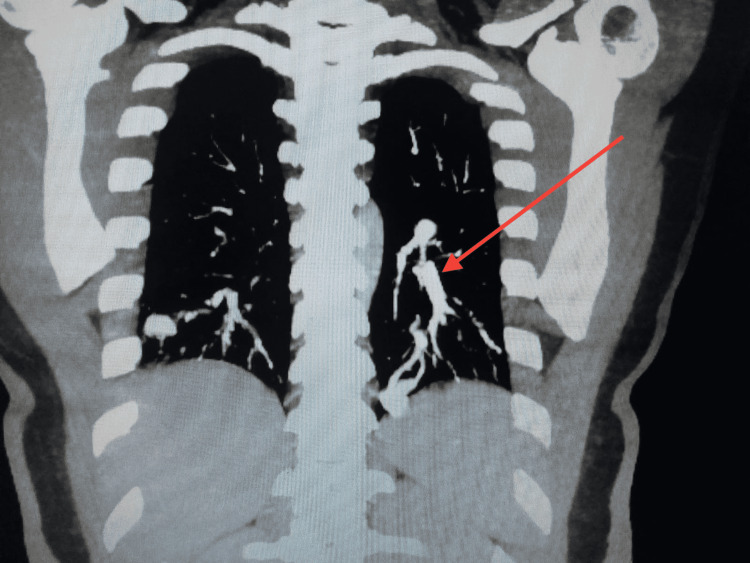
Coronal contrast CT image showing arteriovenous (AV) malformations Coronal contrast reformatted CT image, set on the mediastinal window. Maximum intensity projection (MIP) software was applied to the images to make the vascular connections more conspicuous.

In March 2022, he underwent an EGD in HMC Peshawar, which showed multiple arteriovenous malformations (AVMs) in the fundus of the stomach and multiple oozing AVMs in the second part of the duodenum. At least four abnormal tortuous vessels with highly vascular nidus were seen involving both lower lobes of the lungs (Figure 1).

**Figure 2:**
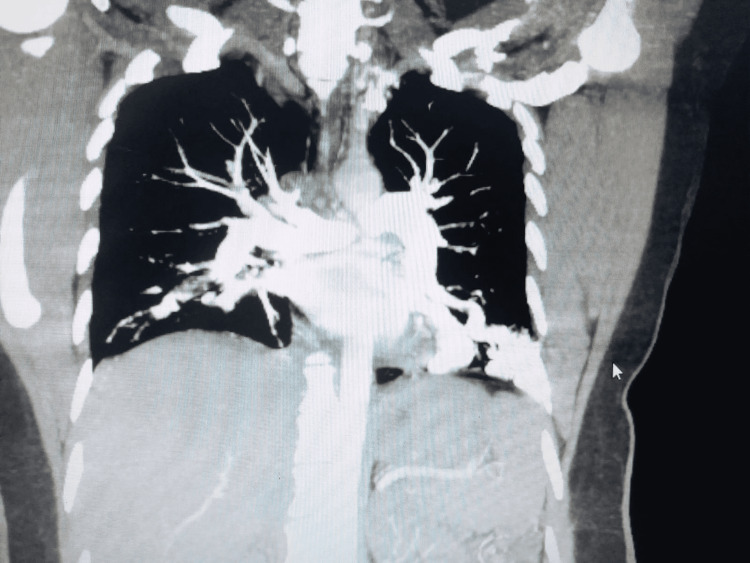
Coronal contrast CT image showing arteriovenous (AV) malformations Coronal contrast reformatted CT image, set on the mediastinal window. Maximum intensity projection (MIP) software was applied to the images to make the vascular connections more conspicuous.

Almost all of these tortuous vessels are supplied by branches of the lower lobe pulmonary artery with their veins draining into the inferior pulmonary veins. The largest one in the lateral basal segment of the left lower lobe measures approximately 2.5 x 2.0 cm (Figure 2). No adjacent area of ground-glass attenuation was identified to suggest pulmonary hemorrhage.

Overall imaging findings are in favor of multiple pulmonary AVMs as detailed above. A spectrum of findings is seen in hereditary hemorrhagic telangiectasia (Osler-Weber-Rendu syndrome). Based on consensus criteria for HHT, the patient was labeled as a definitive HHT patient, as three out of four criteria were met, i.e. (1) history of spontaneous and recurrent epistaxis, (2) pulmonary AVMs and GI AVMs, (3) The father of the patient had a similar history of recurrent epistaxis, and his imaging study showed cerebral AVMs, pulmonary AVMs, and GI AVMs, and he was managed according to the HHT protocol.

Mucocutaneous telangiectasia was not evident in our patient. Based on these criteria, a definitive diagnosis was made and further genetic testing was not done. The patient was started on I/V corticosteroids but did not respond, and hence was started on thalidomide up to 100 mg daily for six weeks. Despite good compliance for seven months, no improvement was seen regarding control of epistaxis, GI bleed, and improvement in blood profile. The patient, during that time, also complained of drowsiness and constipation as major symptoms associated with this drug, and no other side effects related to thalidomide administration were found.

Therefore, it was decided to start him on bevacizumab, 300 mg/week for six doses. On follow-up, by the end of his second dose, the patient's symptoms significantly improved, and his Hb, which upon arrival was 3 g/dl, had improved to 7 g/dl.

## Discussion

Osler-Weber-Rendu syndrome, also called hereditary hemorrhagic telangiectasia (HHT), is a disorder showing an autosomal dominant inheritance pattern resulting in the formation of anomalous vasculature [7]. Clinical features consist of recurrent epistaxis, bleeding from the gastrointestinal tract, and iron deficiency anemia associated with mucocutaneous telangiectasia, i.e., on the lips, oral mucosa, and fingertips. Around one-third of HHT patients have been shown to have hepatic AVMs, which is an increasing concern in these patients [8]. Most AVMs present during childhood are mostly located in the hepatic vasculature followed by the pulmonary and cerebral vasculature in descending order [9]. Bleeding diathesis usually presents during adulthood, mostly after 40 years; around one-third of patients with gastrointestinal bleeding presented with starting symptoms of anemia [10].

The mode of inheritance for HHT is an autosomal dominant pattern that showed variable penetrance and expression. HHT is subdivided into two major types, i.e., HHT1 and HHT2, based on the gene mutated, i.e., endoglin and ALK-1, respectively [11].

About one in 5000 people in North America has HHT, but the peak occurrence was seen in the Afro-Caribbean regions of the Dutch Antilles and France [12-13]. Furthermore, the HHT1 subtype was more prevalent in North America and Europe while HHT2 is common in the Mediterranean and South American regions.

The diagnosis of HHT is made by globally accepted curacao criteria or consensus criteria. The criteria are based on the most prominent features of HHT, i.e., (1) recurrent spontaneous epistaxis, (2) family history of HHT, (3) mucocutaneous telangiectasia, and (4) internal organ involvement [3]. For a definitive diagnosis, at least three out of four features should be present in a patient. The criteria above are the basis for a diagnosis of HHT and give an indication for genetic testing to confirm a diagnosis, i.e., if two out of four features are present in HHT-affected patients, subsequent genetic testing is indicated [14]. Genetic testing is done for the two most commonly mutated genes, i.e., endoglin and ALK-1. After the confirmatory or assumed diagnosis, patients are screened for internal organ lesions. Other disease features include Doppler ultrasound in hepatic AV malformations, transthoracic echocardiography with contrast and agitated saline for pulmonary AVMs, endoscopy for gastrointestinal AV malformations, brain MRI for cerebral AV malformations, and the annual haemoglobin results of all patients [14].

In the case of active nose bleed (epistaxis) in HHT patients, local pressure is applied along with nasal packing and cauterization [15]. These techniques help in effective control of anterior bleed from the Keisselbach plexus but may be ineffective for severe active posterior bleeding, which might need surgical treatment. In the case of life-threatening epistaxis, it was reported that endovascular treatment was an effective approach [15]. The application of humidifiers and moisturizers to nasal mucosa has also been shown to decrease the severity of bleeding effectively [15]. The use of an anti-estrogen such as tamoxifen 20 mg/day has produced a good outcome, as reported from multiple anti-estrogen trials on HHT patients. Trials of tranexamic acid at a dose of 3 g/day have reported a decreased interval of average bleeding daily compared to patients on placebo drugs. However, certain common side effects are reported in these patients such as vertigo and diarrhea. Bevacizumab, an inhibitor of vascular endothelial growth factor (VEGF), can be used intravenously as an intranasal spray or submucosal injection. It has been reported to effectively reduce the epistaxis severity score when given intranasally at a dose of 100 mg. Antiangiogenic and immunomodulating drugs, such as thalidomide and lenalidomide, have decreased episodes of epistaxis. However, its multiple side effects, such as drowsiness, peripheral neuropathy, nausea, and constipation, have limited its use.

For gastrointestinal lesions, repeated endoscopic ablations are done to control bleeding for the short term but show poor outcomes compared to non-HHT patients. Conservative management is required in case of iron-deficiency anemia, supplementing the patient with 35 mg of ferrous gluconate daily. In the case of pulmonary AV malformations, embolotherapy is considered, which shows a decrease in death and severity of the disease. Surgical intervention is necessary for patients where embolotherapy is not indicated. Liver transplantation is considered in patients with end-stage liver disease associated with complicated hepatic AV malformations in HHT patients. Bevacizumab has been considered in patients suffering from end-stage hepatic failure with a lack of lung transplantation facilities and in young patients who are not candidates for liver transplants.

HHT-affected patients have shown a decrease in their life expectancy, but this is mostly dependent on the severity of the disease. Patients showing no internal organ involvement, such as AVMs, in hepatic, pulmonary, and cerebral vasculature have shown normal to near-normal life expectancy as those without HHT. Nearly 10% of patients may die or show severe morbidity related to vascular complications.

## Conclusions

HHT or hereditary hemorrhagic telangiectasia is a rare autosomal dominant disorder that leads to abnormal blood vessel formation in the skin, mucosa, and organs like the liver, lung, and brain. The most common complaint is recurrent epistaxis and intermittent gastrointestinal bleeding (hematemesis or melena), leading to iron-deficiency anemia. Patients with HHT have been treated with recurrent blood transfusions, electrocautery, laser, arterial embolization, and ligation, which are all palliative management. Our patient was treated with multiple blood transfusions and oral thalidomide for recurrent anemia. Therapy with intravenous bevacizumab caused a dramatic reduction in the number of required transfusions and improved blood counts. Although multiple drugs have been evaluated, there is currently no randomized controlled trial confirming efficacy for preventing bleeding from vascular malformations. Our study shows that a clinical trial with intravenous bevacizumab for patients with HHT is warranted.
